# Lunar Tractive Forces and Renal Stone Incidence

**DOI:** 10.1155/2011/813460

**Published:** 2011-10-19

**Authors:** Spyridon Arampatzis, George N. Thalmann, Heinz Zimmermann, Aristomenis K. Exadaktylos

**Affiliations:** ^1^Department of Emergency Medicine, University of Bern, 3011 Bern, Switzerland; ^2^Department of Urology, University of Bern, 3011 Bern, Switzerland

## Abstract

*Background.* Several factors are implicated in renal stone formation and peak incidence of renal colic admissions to emergency departments (ED). Little is known about the influence of potential environmental triggers such as lunar gravitational forces. We conducted a retrospective study to test the hypothesis that the incidence of symptomatic renal colics increases at the time of the full and new moon because of increased lunar gravitational forces. *Methods.* We analysed 1500 patients who attended our ED between 2000 and 2010 because of nephrolithiasis-induced renal colic. The lunar phases were defined as full moon ± 1 day, new moon ± 1 day, and the days in-between as “normal” days. *Results.* During this 11-year period, 156 cases of acute nephrolithiasis were diagnosed at the time of a full moon and 146 at the time of a new moon (mean of 0.4 per day for both). 1198 cases were diagnosed on “normal” days (mean 0.4 per day). The incidence of nephrolithiasis in peak and other lunar gravitational phases, the circannual variation and the gender-specific analysis showed no statistically significant differences. *Conclusion.* In this adequate powered longitudinal study, changes in tractive force during the different lunar phases did not influence the incidence of renal colic admissions in emergency department.

## 1. Introduction

Kidney stone formation is usually due to environmental, metabolic, or genetic factors. A precise causative factor is not identified in most nephrolithiasis patients. A family history of kidney stones, insulin resistance, hypertension, obesity, low urine volume, and dietary habits is associated with an increased risk [1]. Stone disease also shows marked geographic variability, and the phenomenon of the “stone season” has been attributed to both increased environmental temperatures and sunlight levels [2]. The expected environmentally related increase in the prevalence of nephrolithiasis can cause a significant increase in renal colics admissions, with the corresponding strain on healthcare delivery and related costs [3]. Apart from this well-known risk factor, little is known about the impact of other potential triggers on nephrolithiasis, such as lunar gravitational forces.

The lunar phases have been held responsible for affecting human health since antiquity. The effect of the lunar phases on human physiology is well documented, and relevant studies have shown an associated increase in seizures, deregulation of the cardiovascular system, aggressive behavior, changes in menstruation, and spontaneous full-term deliveries [4–8]. Studies concerning the lunar gravitational effect on renal colics frequency are scare and no adequately powered longitudinal study has ever explored this relationship [9]. 

To test the hypothesis that the incidence of symptomatic renal colics increases at the time of the full and new moon because of increased lunar gravitational forces, we conducted a retrospective study and reviewed the frequency of admission of patients with renal colics to our emergency department over the last 11 years and compared this with the corresponding lunar phases.

## 2. Materials and Method

We collected and retrospectively analyzed all data on patients (>18 years old) attending the emergency department of the Inselspital in Bern (Switzerland) between 2000 and 2010 due to renal colics. Our tertiary care facility is the only “level one” unit in this area, covering a population of about 1,000,000 people and seeing about 28,000 adult patients per year. We included only patients with renal colics in whom nephrolithiasis was diagnosed using non-contrast-enhanced spiral abdominal computed tomography, abdominal sonography, or i.v. urography. Clinical and radiological data were taken from the department's computerized case database, which records data on all new admissions. Admissions between 1 January 2000 and 31 December 2010 were analyzed, and the mean daily number of nephrolithiasis cases was compared with the corresponding lunar phase day. During one lunar orbit, the phase in which the moon is fully illuminated, from the perspective of the earth, is called full moon. It is defined as three-day period in the 29.5-day lunar cycle, with the middle day being designated as the full moon. New moon is the lunar phase when moon is first visible as seen from earth. In our analysis, the dates of full and new moon days were grouped into a minimum of 3 days per phase. The lunar phases were defined as full moon ±1 day, new moon ±1 day, and the days in-between as “normal” days, based on data on lunar phases from the U.S. Naval Observatory Astronomical Applications Department [10]. The incidence of renal colic events was calculated as mean cases number per lunar phase day. 

## 3. Results

**Figure 1:**
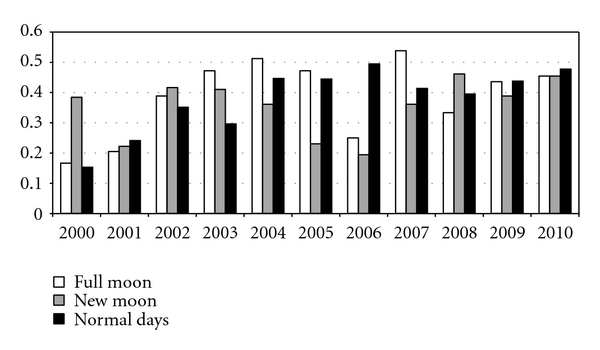
Mean episodes of renal colics per day in relation to lunar phases in 1,500 patients over an 11-year period (2000–2010).

1,500 consecutive nephrolithiasis patients (1,152 males and 348 females) were admitted during the 11-year observation period (Figure 1), with a mean daily admission rate of 0.4. During this period, 156 cases of acute renal colics were diagnosed at the time of a full moon and 146 at the time of a new moon (mean of 0.4 per day for both). 1198 cases were diagnosed on “normal days” (mean also 0.4 per day). The differences between the mean rates of renal colics at the time of a new moon, full moon and on other days of the lunar cycle were not statistically significant. Since the data were collected over an about 10-year span, there may also be a circannual variation that when unassessed could obscure a lunar effect. There may have been added circadecadal and even other longer-term changes from one year to another, and, if so, it may be interesting to find out whether a lunar effect could be detected during some years but not during other years. In order to better analyze the circannual variation, a yearly analysis of the data was undertaken which showed no significant differences between the mean rates of renal colics. Also, since for males and females are precedents for gender differences in the incidence of pathology of stone disease, we undertook a gender-specific analysis of our data. Due to the small women sample of our study only men data were analyzed showing no significant differences between the mean rates of renal colics during the observational period.

## 4. Discussion

This adequate powered longitudinal study shows that no positive relationship appears to exist between the different lunar phases and the incidence of acute renal colic admissions in the emergency department. 

Pytheas of Massalia, a Greek explorer who circumnavigated Britain, was first to associate the tides with the lunar phases. In modern gravitational theory, as the Moon orbits the Earth, it aligns with the Earth and Sun semimonthly during the full moon and new moon phases. At such times, we have extremely high tides [11]. The cosmic interaction with the Moon has a relatively large impact on the surface of the earth. The gravitational force exerted by the Moon depends on its position in relation to the Sun and the Earth. The “tractive” or tide-producing force of the Moon on the Earth is defined by Newton's law of gravity, which means that the force is proportional to the mass of the Moon and inversely proportional to the square of the distance between Moon and Earth. These effects are superimposed on the Earth's gravitational force and draw the ocean waters toward positions beneath these external celestial bodies and, because of the centrifugal force of the Earth's motion, also toward the opposite position. On the other hand, the gravitational force on the human body is greater than the tractive force, by a factor of roughly 300,000. Thus, the effects of tractive forces on the human body do not represent a physiological stimulus of renal colic and no relationship appears to exist between the lunar phases and acute renal colic admissions.

This study has certain limitations. First, this was a retrospective study from a single hospital since Januar 2000. However, our study covers a 11-year period and is adequately powered with a cohort of 1500 radiographically confirmed stone patients. Additionally, our sample included only a comparison among 3 time spans (full moon, 3 days bracketing new moon, and on remaining days) but the time of admission was not recorded. A time series analysis could provide additional information regarding the presence or absence of an about 29-day cycle or the presence of any other rhythmic behavior that may have obscured the results.

## 5. Conclusion

The Moon will continue to influence some natural phenomena, but our findings strongly support the theory that the Moon's tractive forces do not influence the incidence of nephrolithiasis.
